# Intrapleural Fibrinolytic Therapy With Alteplase for the Management of Multiloculated Malignant Pleural Effusion: A Case Series

**DOI:** 10.7759/cureus.27549

**Published:** 2022-08-01

**Authors:** Marko Nemet, Milica Vasilić, Sanja Ergelašev, Ivan Kuhajda, Ivan Ergelašev

**Affiliations:** 1 Internal Medicine, Faculty of Medicine, University of Novi Sad, Novi Sad, SRB; 2 Cardiology, Institute for Cardiovascular Diseases of Vojvodina, Sremska Kamenica, SRB; 3 Thoracic Surgery, Institute for Pulmonary Diseases of Vojvodina, Sremska Kamenica, SRB; 4 Surgery, Faculty of Medicine, University of Novi Sad, Novi Sad, SRB

**Keywords:** chest tube drainage, pleural malignancy, loculated pleural effusion, alteplase, intrapleural fibrinolytic therapy, malignant pleural effusion

## Abstract

Malignant pleural effusion refers to the presence of fluid in the pleural space due to an underlying malignancy. Malignant pleural effusion is sometimes accompanied by the formation of fibrous adhesions resulting in a multiloculated effusion. This diminishes the efficacy of drainage and makes successful pleurodesis impossible, leaving the patients with severe shortness of breath. In the process of freeing the pleural space from fluid-filled loculations, intrapleural application of fibrinolytic is being investigated as a possible therapeutic approach. Here, we report four cases of adult patients hospitalized for malignant pleural effusions who were treated with intrapleural fibrinolytic therapy at the Institute for Pulmonary Diseases of Vojvodina, Republic of Serbia.

## Introduction

Malignant pleural effusion (MPE) refers to the presence of fluid in the pleural space due to an underlying malignancy [1]. The leading causes of MPEs are lung cancer, breast cancer, and lymphomas. Any metastatic lesion in the pleural space followed by the destruction of lymphatic vessels could lead to a decrease in absorption and subsequent accumulation of the fluid in the pleural space [2]. Additionally, malignancy-induced inflammation leads to increased vascular permeability and higher rates of pleural fluid production. As procoagulant and fibrinolytic activities are usually imbalanced, MPEs are, in some patients, accompanied by the formation of fibrous adhesions resulting in a multiloculated effusion. This diminishes the efficacy of drainage and makes successful pleurodesis impossible, leaving the patients with severe shortness of breath, which limits their quality of life [3]. In the process of freeing the pleural space from fluid-filled loculations, intrapleural application of fibrinolytics is being investigated as a possible therapeutic approach [3]. MPE often portends a poor prognosis and has a detrimental effect on life quality. Thus, palliative care and symptomatic relief are of utmost importance in these patients [4,5].

Here, we report four cases of adult patients hospitalized for MPEs who were treated with intrapleural fibrinolytic therapy (IPFT) at the Institute for Pulmonary Diseases of Vojvodina, Republic of Serbia, in the time period of January 2019 to January 2020. The Institute for Pulmonary Diseases of Vojvodina is a tertiary healthcare institution that provides healthcare to two million people living in Vojvodina. This case series reflects on intrapleural fibrinolytic therapy as a palliative therapeutic approach for loculated MPEs.

## Case presentation

Case 1

**Figure 1:**
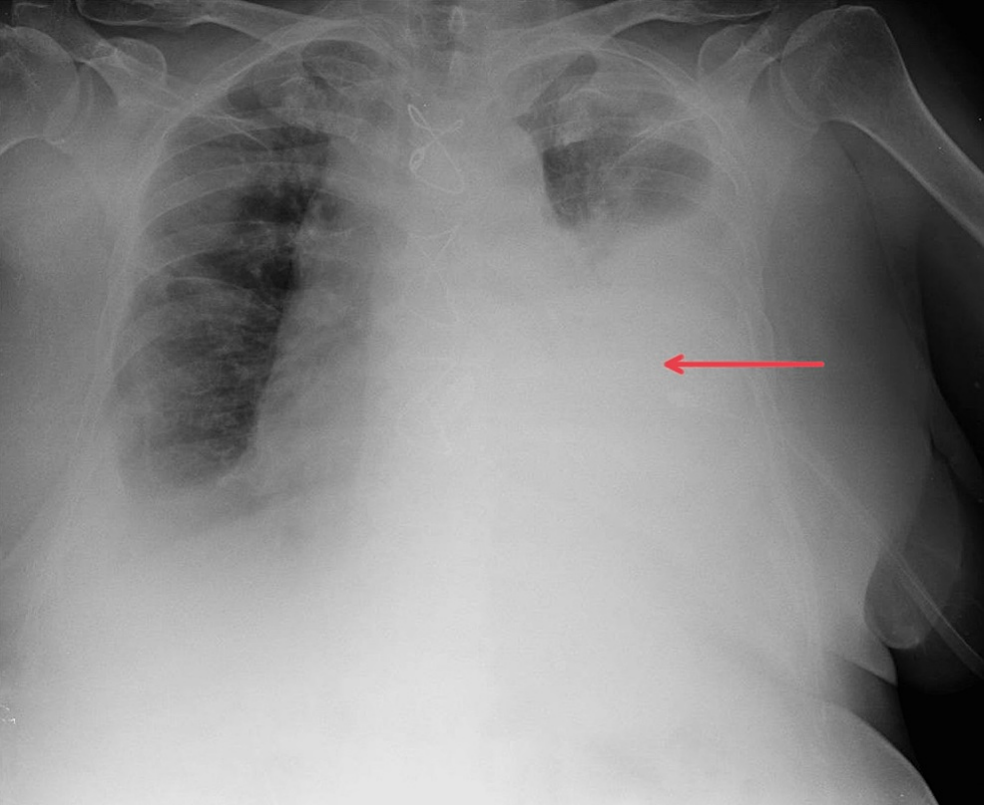
Chest X-ray of patient 1 prior to intrapleural fibrinolytic therapy The red arrow shows a large left-sided pleural effusion.

The patient was a 65-year-old woman diagnosed with multiple myeloma. She presented with shortness of breath. Chest X-ray (CXR) showed a massive left-sided pleural effusion (Figure 1), and the patient was admitted for drainage and pleurodesis. Myeloma plasma cells were identified on pleural fluid cytology. The patient’s Eastern Cooperative Oncology Group (ECOG) performance status before drainage was two. A chest tube was placed, which initially resulted in 2100 mL of serosanguineous fluid during the first 24 hours. On the second day of hospitalization, the drainage ceased but the patient still had shortness of breath. Chest computed tomography (CT) showed multiloculated effusion and, therefore, the patient met indications for IPFT. Application of 5 mL of alteplase was followed with drainage of 1300 mL serous fluid in the first 24 hours, after which the second 5 mL dose of alteplase was administrated. No more fluid was drained in the next 48 hours. The patient reported a significant subjective improvement. CXR showed complete re-expansion of the lung and in two days, the chest tube was removed. Pleurodesis was contraindicated since pleural fluid analysis showed low pH value and low glucose levels, which represented a high risk for the development of empyema. There were no adverse effects from the IPFT and the patient was discharged six days after admission. Five days after discharge the patient returned presenting with shortness of breath. CXR showed reaccumulation of fluid in the pleural space and 550 mL of fluid was subsequently drained after which she was discharged. The patient passed away ten months later.

Case 2

**Figure 2:**
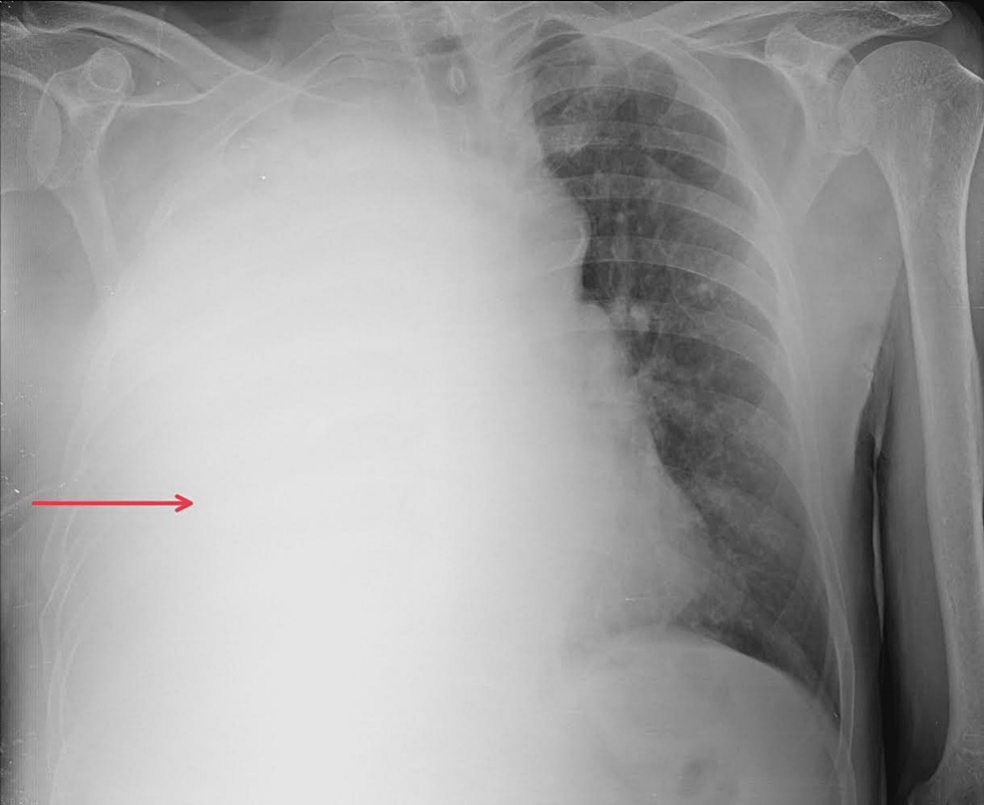
Chest X-ray of patient 2 prior to intrapleural fibrinolytic therapy The red arrow shows a near-complete right hemithorax white-out secondary to a massive pleural effusion.

**Figure 3:**
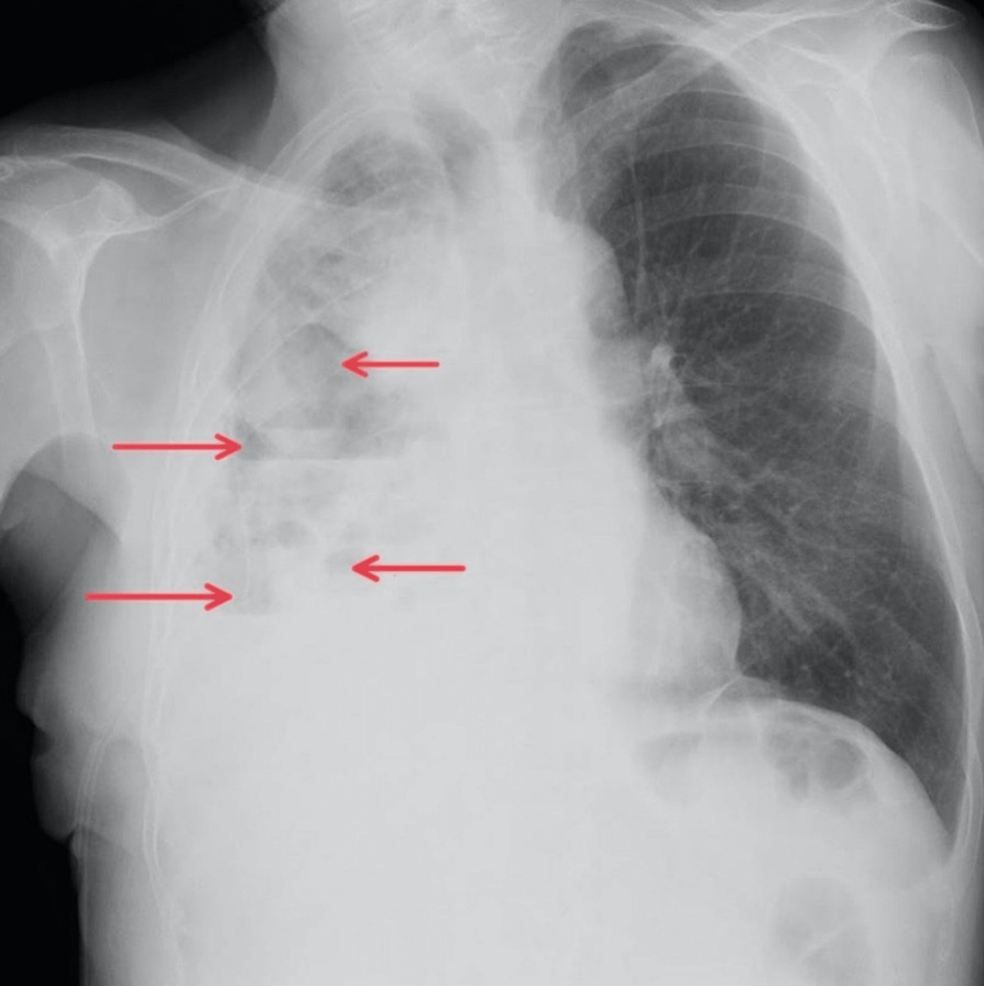
Chest X-ray of patient 2 after intrapleural fibrinolytic therapy Red arrows show areas of increased lucency in the right hemithorax with several air-fluid levels, 72 hours after intrapleural fibrinolytic therapy.

The patient was a 70-year-old man diagnosed with lung adenocarcinoma, T2bN3M1a, stage IVa. He presented with severe shortness of breath and dull chest pain. A large right-sided MPE was seen on CXR that required drainage (Figure 2). His ECOG performance status before drainage was three. Initially, 2900 mL of serosanguineous fluid was drained. Three days later, drainage stopped, but pleural effusion was still observed on CXR. Pleural septations and loculations were confirmed on ultrasound. Therefore, as he met indications for IPFT, 2.5 mL of alteplase was administrated. Shortly after, he reported severe, sharp chest pain that subsided quickly. During the first 24 hours after IPFT administration, 2600 mL of serous fluid was drained. An additional 900 mL of serosanguinous fluid was drained on the second day. Also, a small amount of air was noted during the drainage. On the third day after IPFT, CXR showed a radiological improvement (Figure 3). Even though the patient did not report a significant subjective improvement, he was discharged with a Heimlich valve since an indwelling pleural catheter (IPC) was unavailable. Pleurodesis was not performed since no adequate apposition of pleural membranes was seen on CXR. On the follow-up, one month later, the patient reported that 300 to 700 mL of serosanguineous fluid was draining per day. CXR showed partial re-expansion of the right lung with several air-fluid levels in the pleural space but without a significant effusion. Two months after the follow-up, the patient passed away.

Case 3

**Figure 4:**
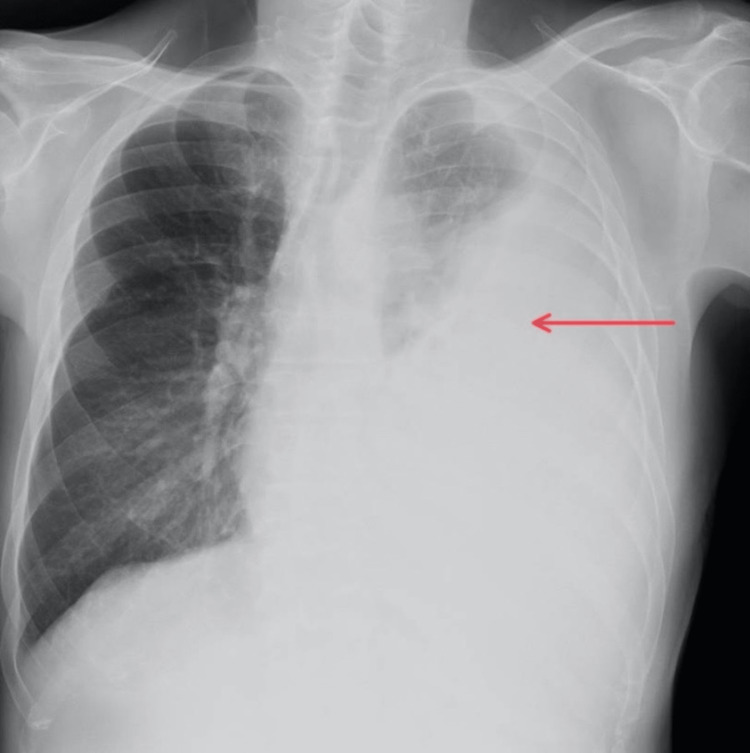
Chest X-ray of patient 3 prior to intrapleural fibrinolytic therapy The red arrow shows a large left-sided pleural effusion.

The patient was a 60-year-old man diagnosed with small cell lung cancer, T4N3M1a, stage IVa. He presented with shortness of breath. CXR showed left-sided MPE (Figure 4), and a chest tube was placed. His ECOG performance status before drainage was two. Initially, 1000 mL of serous fluid was drained. As shortness of breath persisted, chest CT was performed and pleural loculations were observed. Therefore, 5 mL of alteplase was given as IPFT. Second and third doses of alteplase were given, 24 and 48 hours later, respectively. This time, the dose was reduced to 2.5 mL. In the first 48 hours following IPFT, 2700 mL of serous fluid was drained. After 48 hours, the fluid became serosanguineous and the air started to leak. Ten days later, clinical and radiological improvements were noted. However, as there was no adequate apposition of pleural membranes on CXR, the patient was discharged with a Heimlich valve since IPC was unavailable. Ten days after discharge, CXR showed complete obliteration of pleural space, and the Heimlich valve was removed. The patient did not experience reaccumulation of MPE and was able to undergo six cycles of chemotherapy and one cycle of radiation therapy. Nine months after discharge from our institution, he passed away.

Case 4

**Figure 5:**
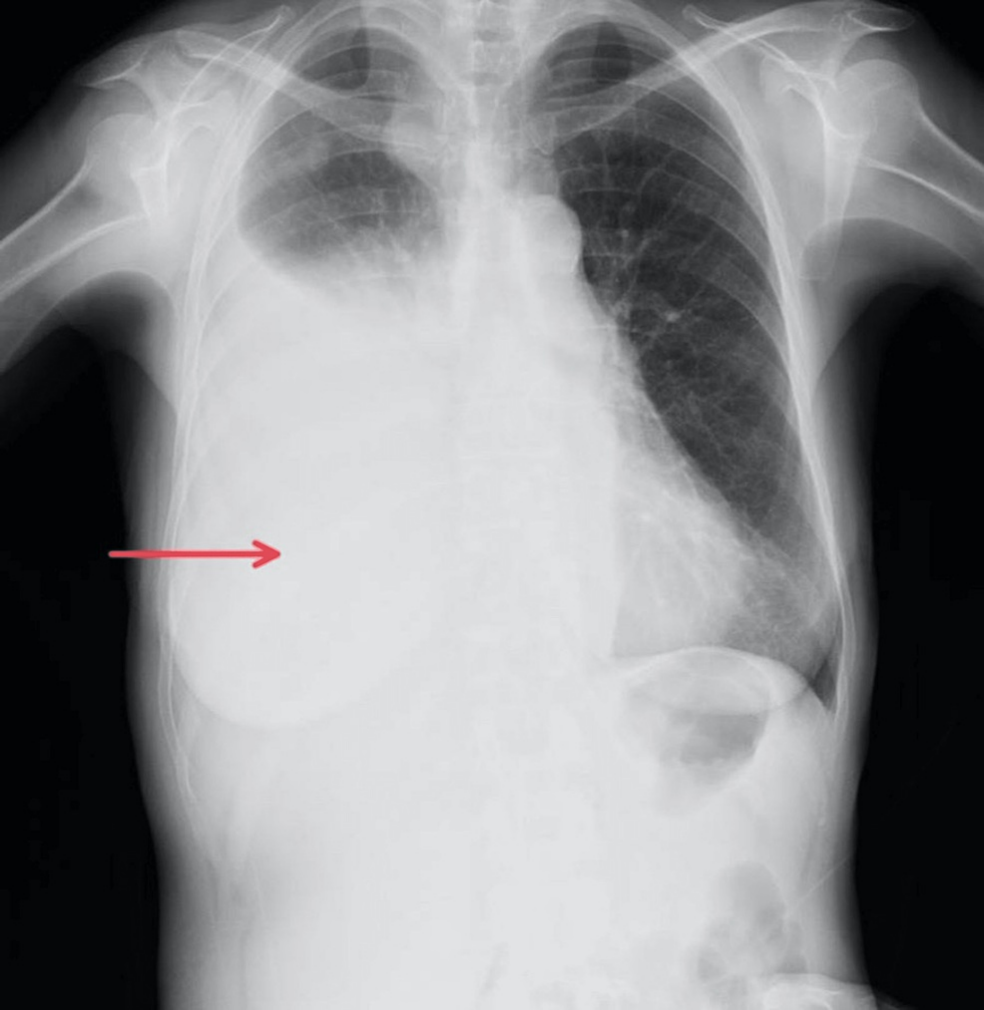
Chest X-ray of patient 4 prior to intrapleural fibrinolytic therapy The red arrow shows a large right-sided pleural effusion.

**Figure 6:**
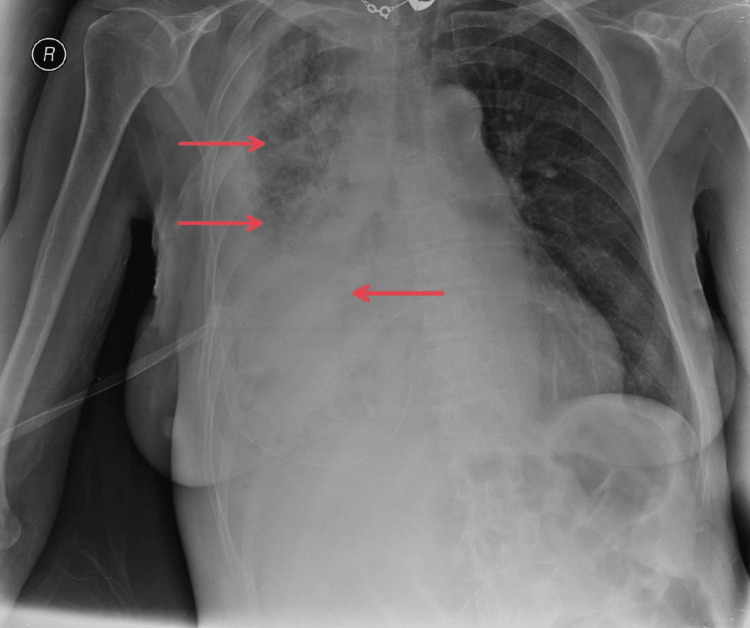
Chest X-ray of patient 4 after intrapleural fibrinolytic therapy Red arrows show areas of increased lucency in the right hemithorax, 72 hours after intrapleural fibrinolytic therapy.

The patient was a 61-year-old woman diagnosed with lung adenocarcinoma, T2aN1M1a, stage IVa. She presented with severe shortness of breath. On CXR, right-sided MPE was noted (Figure 5) and chest tube drainage was initiated. Her ECOG performance status before drainage was three. In the first two days, 2700 mL of serous fluid was drained. As adequate apposition of pleural membranes was observed, pleurodesis with 2 g of talc slurry in 100 mL of isotonic saline was performed. After pleurodesis, continuous chest tube drainage of up to 100 mL fluid daily prompted ultrasound investigation. Subsequently, multiloculated effusion was noted. Thus, as IPFT was indicated, 5 mL of alteplase was administered and an additional 5 mL of alteplase was given the next day. IPFT resulted in 650 mL of serous fluid during the first 72 hours after IPFT. Later, the fluid became serosanguineous and frank blood was also noted. There was a radiographical improvement (Figure 6). Nevertheless, as the patient was severely ill, no clinical improvement was observed. The patient passed away a week later.

## Discussion

The treatment of MPE consists of pleural drainage and pleurodesis. Chest drainage aims to remove the fluid and expand the lungs while pleurodesis helps prevent MPE recurrence. As this is a palliative treatment, the main goal is to relieve symptoms and improve the quality of life. Some pleural effusions are resistant to chest tube drainage resembling a true challenge in management. In some patients, radiological investigation reveals multiple loculations in the pleural space due to fibrinous adhesions. This renders the lungs unable to fully expand which is a prerequisite for successful pleurodesis. To efficiently drain loculated pleural effusion, IPFT was recently introduced as a promising therapeutic approach [1-5].

Fibrinolytic agents were first used to enhance drainage in complicated parapneumonic effusions and empyema [6,7]. As IPFT showed positive effects in these cases, the question arose whether it could be used in loculated MPEs. The hypothesis is that IPFT causes lysis of the fibrinous bands and thereby enhances drainage and lung re-expansion. This would subsequently lead to adequate apposition of pleural membranes, which is needed for successful pleurodesis [8].

The fibrinolytic agent used in the above-mentioned cases was alteplase. After the failure of chest drainage, observed residual pleural effusion on CXR, persistent symptoms, and/or continuous drainage after talc pleurodesis, loculated MPE was confirmed via other imaging modalities, such as chest CT or ultrasound. This was the indication for IPFT. In other studies, streptokinase and urokinase were the agents most often used. The indications for IPFT in these studies were similar to ours [8-11]. In our cases, we used different doses of alteplase. We initially followed our institutional protocol for administrating alteplase in case of empyema which states that 5 mL of alteplase in 50 mL of normal saline is instilled via a pleural chest tube. The chest tube is clamped for four hours following IPFT administration. The same dose of alteplase can be administrated up to three times with at least 24 hours apart. The patient number one and number four were the first to be treated according to this protocol, hence they received two 5 mL doses of alteplase. However, after the fourth patient experienced hemorrhagic drainage fluid, we decreased the second and the third dose to 2.5 mL. This was the case in our third patient who received three doses of alteplase in total. Even though the second and the third dose were reduced, this patient experienced air leak following IPFT. Therefore, we came to conclusion that the 5 mL dose of alteplase was too high for our cancer patients, thus we further reduced all doses of alteplase to 2.5 mL.

A study by Davies et al. was one of the first to show the positive effects of IPFT on multiloculated MPEs [9]. Their study showed that intrapleural administration of streptokinase in radiologically confirmed loculated effusion resulted in increased drainage output, radiographic improvement, and relief of shortness of breath. Successful pleurodesis was performed in 40% of patients and no effusion re-accumulation was observed. More recent studies by Hsu et al. and by Saydam et al. confirmed these results by comparing patients that underwent IPFT with a control group where IPFT was not performed [10,12]. Both studies found a significant radiographic improvement, higher drainage volumes, as well as symptomatic relief in the IPFT groups. Okur et al. found significant radiographic improvement and higher drainage volumes following intrapleural streptokinase administration [8]. In contrast to these, a study by Mishra et al. showed only a significant radiographic improvement without better pleural drainage or a change in clinical status [11].

Even though IPFT did not lead to adequate pleural membranes apposition for pleurodesis to be performed, all of our patients showed some degree of radiographical improvement. Three out of four patients had improved drainage after IPTF administration. The only patient that did not have higher drainage volumes after IPFT had previously undergone pleurodesis. Newly formed adhesions from pleurodesis could explain the failure to increase drainage volumes. Regarding clinical status and improvement in symptoms, two out of four patients experienced substantial relief of shortness of breath. The other two patients failed to improve clinically.

In a study by Hsu et al., 59.2% of patients who received IPFT underwent pleurodesis after successful intrapleural fibrinolysis. However, the success rate of pleurodesis was similar between the patients in the simple pleurodesis group who did not receive IPFT and the patients in the IPFT group [13]. Mishra et al. showed no difference in time to pleurodesis failure in the IPFT group and the placebo group [11]. Unfortunately, we failed to perform pleurodesis after IPFT in our patients. Reasons were inadequate apposition of pleural membranes, high risk of empyema, and/or poor clinical status. Nevertheless, the third patient experienced "auto-pleurodesis" as his pleural space spontaneously obliterated, which resulted in a removal of the Heimlich valve. In the first and second patients, reaccumulation of effusion was noted. The last patient died soon after IPFT.

Since MPE usually represents advanced-stage malignancy, the median survival time is estimated to be between three and 12 months. This depends on age, tumor type, time of diagnosis, stage of the malignancy, performance status, and response to systemic therapy [14]. It is worth mentioning that our patient from case number three was able to continue with the treatment plan for the underlying small cell lung cancer after his pleural space spontaneously obliterated. He lived nine more months after MPE occurred. Mishra et al. found that IPFT decreased mortality. This decrease, even though small, was significant. They also showed a shorter hospital stay in patients that underwent IPFT [11]. Similarly, a study by Hsu et al. in 2006 demonstrated a shorter duration of chest tube drainage after fibrinolytic administration [12]. In a study by Hsu et al. in 2019, IPFT did not have an impact on mortality, per se. However, their study showed that successful pleurodesis, with or without IPFT, prolonged survival. IPFT followed by pleurodesis, on the other hand, resulted in a longer hospital stay [13].

Although rare, the most common reported side effect of IPTF is acute, sharp chest pain [8,9,11]. Additionally, Hsu et al. reported serious side effects of IPFT in 2.2% of subjects. [13]. These were hemothorax in three patients, and *Pseudomonas aeruginosa *empyema in one. Mishra et al. also reported two pleural space infections, one in the IPFT group and one in the placebo group [11]. In our cases, only one patient experienced acute chest pain shortly after IPFT administration. After adequate analgesic medication, his pain subsided and the patient did not report pain recurrence. Two of our patients had air leaks during the pleural drainage after IPFT. The air leaks were small and did not last for long. All of our patients had serosanguinous drainage after IPFT. This finding could be attributed to direct vessel damage by the IPFT and/or by the nature of MPE itself. Besides that, in one patient, frank blood was drained after 72 hours from IPFT initiation. In contrast to others, however, this patient received substantially higher doses of IPFT in a short time interval.

## Conclusions

Radiographic improvement and symptomatic relief after IPTF were observed in our four cases. Thus, we suggest considering IPFT as a potential therapy for loculated and hard-to-drain MPEs. This is important when palliative treatment options are limited, especially when patients are suffering from severe shortness of breath. In the light of our results, we would advise further research on IPFT as an approach to loculated MPEs. Namely, it is important to establish proper protocols, such as the right fibrinolytic agent, dosages, and dosing intervals, as well as indications and contraindications for IPFT in patients with MPEs.
